# Complexity galore: 3D cultures, biomechanics and systems medicine at the eighth ENBDC workshop “Methods in Mammary Gland Development and Cancer”

**DOI:** 10.1186/s13058-016-0777-2

**Published:** 2016-11-25

**Authors:** Bethan Lloyd-Lewis, Anoeska A. A. van de Moosdijk, Mohamed Bentires-Alj, Robert B. Clarke, Renée van Amerongen

**Affiliations:** 1 Department of Pathology, University of Cambridge, Cambridge, CB2 1QP UK; 2 Section of Molecular Cytology and Van Leeuwenhoek Centre for Advance Microscopy, Swammerdam Institute for Life Sciences, University of Amsterdam, Science Park 904, 1098 XH Amsterdam, The Netherlands; 3 Friedrich Miescher Institute for Biomedical Research (FMI), Maulbeerstrasse 66, CH-4058 Basel, Switzerland; 4 Division of molecular and clinical cancer sciences, Manchester Cancer Research Centre, University of Manchester, Wilmslow Road, Manchester, M20 4QL UK

## Abstract

The ENBDC workshop “Methods in Mammary Gland Development and Cancer” is an established international forum to showcase the latest technical advances in the field. The eighth meeting focused on emerging concepts and technologies for studying normal and neoplastic breast development.

## Introduction and keynote

Along the shores of Lake Lucerne scientists working on breast development and cancer gathered in Weggis for the annual European Network for Breast Development and Cancer (ENBDC) workshop. The uncharacteristic downpours failed to dampen the enthusiasm of participants, fuelled by high-caliber talks and the exchange of cutting-edge insights relevant to mammary gland biology. This year’s keynote lecture was given by Jos Jonkers (Netherlands Cancer Institute, Amsterdam, the Netherlands). He discussed how gene targeting in embryonic stem cell lines derived from already existing genetically engineered mouse models, so-called GEMM-ESCs, has greatly improved his lab’s efficiency to generate complex compound mouse models [1]. This allows a relatively rapid in vivo analysis of a gene’s contribution to the breast cancer phenotype, response to therapy, and resistance to treatment. He illustrated this by discussing the effects of oncogenic *Met* expression on top of *Brca1* and *p53* loss [2]. His lab is also experimenting with CRISPR/Cas9 and Jonkers warned that local delivery of Cas9 appears to trigger an immune response, which researchers should bear in mind when applying Cas9 somatically [3].

## Session 1: Systems biology, large-scale approaches and high throughput screening (Chair: Mohamed Bentires-Alj)

Anne-Lise Børresen-Dale (Institute for Cancer Research, Oslo, Norway) described breast tumor heterogeneity as the biggest challenge for translating biological findings into the clinic. Børresen-Dale and collaborators have taken a holistic view and use a patient directed “systems medicine” approach to understand how inter- and intra-tumor heterogeneity affects response to therapy and patients’ outcome [4, 5]. Their multilevel approach includes molecular analyses of breast cancer: assessment of DNA copy number variations, mutation and methylation, as well as alterations in RNAs, microRNAs, long-noncoding RNAs, proteins, and metabolites. It also comprises imaging (i.e., mammograms and CT/MRI/PET) and clinical and pathology-based classification. It remains unclear which levels best capture both the intra- and inter-tumor heterogeneity important for treatment decisions, and algorithms that integrate data from all levels are still missing.

Luca Magnani (Imperial College, London, UK) described how tumors evolve (epi)genetically and linked these alterations to biomechanical changes in the tumor. Long-term estrogen-deprived (LTED) cells that became resistant to aromatase inhibitors (AI) acquire metastatic potential and increase the expression of genes involved in cholesterol biosynthesis. AI-resistant cells upregulate cholesterol biosynthesis and activate estrogen receptor α (ERα) to promote invasion, which can be attenuated with anti-cholesterol treatment. This suggests that a biomarker signature based on cholesterol biosynthesis might be used to stratify patients prior to adjuvant endocrine therapies [6]. Also, the keratin type II locus topological associating domain (TAD) is among the top 5% of hyper-acetylated TADs in LTED AI-resistant cells. Keratin80 (*K80*) is upregulated by cholesterol and was found to be the driver within the TAD. Of note, *K80* is overexpressed in metastatic breast cancers and seems to increase intracellular stiffness. They also identified copy number variation as a potential mechanism of AI resistance, which may synergize with epigenetic reprogramming to drive the development of an estrogen-independent niche within metastatic tissue.

Francesca Buffa (University of Oxford, UK) discussed in silico systems biology and functional genomics approaches to accelerate biomarker discovery. She used in silico co-expression networks to define pathways from human cancer samples and developed “SEARCH”: SEed Agglomerative and Recursive Clustering with Hypothesis oriented initialization. SEARCH exploits knowledge of cancer pathways to construct a gene network of a given cancer phenotype (e.g., hypoxia, angiogenesis) and derive a signature [7]. Signatures were validated in human breast cancer samples and are currently being tested for whether they are generalizable to other tumors.

## Session 2: PhD and postdoc session (Chairs: Bethan Lloyd-Lewis and Anoeska van de Moosdijk)

For the first time in the meeting’s history, the floor was briefly entrusted to the next generation of researchers in the PhD and postdoc session. David Bryant (University of Glasgow, UK) discussed the application of three-dimensional (3D) organoid cultures to investigate collective cancer cell invasion. He provided a historical overview and critical assessment of 3D culture, before presenting the approaches undertaken in his laboratory to study cell polarity and invasion in prostate cancer. Using immortalized and tumor cell lines grown in Matrigel, he showed how the scratchwound assay could be adapted to 3D. Combined with time-lapse imaging, this approach provided high-resolution insights into the role of IQSEC1 (a guanine nucleotide exchange factor for ARF6) in cell invasion, with knockdown cells failing to repopulate the scratched area despite showing protrusion formation (unpublished data). He underlined the importance of studying cell behavior on a population level and is currently developing automated image segmentation for high-throughput analysis of organoid cultures.

## Session 3: Emerging models and technologies (Chair: Renée van Amerongen)

Pekka Katajisto (University of Helsinki, Finland) discussed his search to identify an in vitro system that showed nice asymmetric cell division and shared his eureka moment when he found immortalized human mammary epithelial cells (HMECs), which ultimately allowed him to demonstrate the age-selective segregation of mitochondria [8]. He showed how the SNAP-tag technology [9], which facilitates the attachment of fluorophores to specific proteins in live cells, allows stress-free labeling of differently aged organelles with multiple fluorochromes, enabling pulse-chase experiments while preventing damage to the mitochondria.

Walid Khaled (University of Cambridge, UK) talked about his efforts to elucidate the role of *BCL11A* in triple-negative breast cancer [10]. Using rapid immunoprecipitation mass spectrometry of endogenous proteins (RIME), his team is characterizing the BCL11A interactome. He also discussed the intricacies associated with generating a CRISPR reporter cell line via homology directed repair (HDR), stressing that the targeting efficiency greatly depends on both the cell line and sgRNA design.

Sara Wickström (Max Planck Institute for Biology of Ageing, Cologne, Germany) talked about the regulation of epidermal stem cell fate, stressing the importance of stochasticity. Whereas cells typically show self-organization into a functional tissue, it can be difficult to predict cell fate decisions for individual cells in the population. Her group found that, quite unexpectedly, hair follicle stem and progenitor cells interconvert equally in both directions (Chacon-Martinez et al., submitted). She showed how mechanical strain can influence cell fate decisions by re-arranging the chromatin structure in the nucleus, a process that involves transcriptional silencing by the Polycomb complex [11].

## Session 4: Cell identity and plasticity in the mammary gland (Chair: Rob Clarke)

Christina Scheel (Helmholtz Centre, Munich, Germany) presented on epithelial plasticity in human mammary gland morphogenesis and breast cancer progression. She discussed a novel 3D culture method for normal human breast epithelium [12]. Human breast epithelial cells obtained from reduction mammoplasties were cultured as organoids in floating collagen I gels in medium containing bovine pituitary extract and forskolin. This system enables normal breast epithelial cells to be passaged in vitro and forskolin prevents epithelial–mesenchymal transition (EMT), which otherwise prevents the formation of organoids that resemble Terminal-Ductal lobular units (TDLUs). She also highlighted a model of EMT in breast cells that advances our understanding of its importance in metastasis [13]. Here, hTERT-immortalized human breast epithelial (HMLE) cells were transduced with a tamoxifen-inducible *TWIST* gene (HMLE-Twist-ER). Treatment of HMLE-Twist-ER cells with tamoxifen induces EMT defined by *CD44* expression and mammosphere formation. Subsequent withdrawal of tamoxifen reveals three populations, including CD44^+^CD24^+^ cells, which retain epithelial characteristics and have enriched mammosphere-forming ability. These cells originate from HMLE cells that express TGF-beta receptor type 1 and *WNT5a* and are potentially more active in lung colonization than cells that have transdifferentiated to a mesenchymal state.

Beatrice Howard (Institute of Cancer Research, London, UK) discussed embryonic mammary cell identity and plasticity. It is known that embryonic day 12.5 (E12.5) mammary gland primordia can repopulate a cleared adult mammary fat pad and that mammary-repopulating units increase as the embryonic gland matures. However, the factors regulating this remain unknown. As the organ initially forms, *Hox*, *Wnt*, and stem cell genes such as *Edar* and *Nrg3* are highly expressed. *Nrg3* ^*−/−*^ mice have an embryonic phenotype with absent or hypoplastic mammary organs composed of cells that have not been specified to the mammary phenotype [14]. To facilitate mutant phenotypic studies, several embryonic mammary cell lines have been developed by cloning them out in 3D culture. These have some distinctive phenotypes, including expression of specific adult stem cell markers such as *Sox9* and *Procr* and capacity to form partial post-natal mammary epithelial outgrowths.

## Conclusions

The invited speakers were supported by nine selected short talks, with the first ENBDC DeOme prize for best talk awarded to Colinda Scheele (Hubrecht Institute, Utrecht, the Netherlands). Also, Bethny Morrissey (Leeds Institute of Cancer and Pathology, UK) publicized SEARCHBreast (https://searchbreast.org), a resource that facilitates sharing of material from in vivo breast cancer models [15]. Lively poster sessions and a collegial atmosphere fostered the dissemination of ideas and technical experiences between participants. Overall, the annual ENBDC workshop reaffirmed its position as an essential forum for discussion in the mammary gland and breast cancer field. The ninth ENBDC workshop, on 9–11 March 2017, will be chaired by Richard Iggo (Institut Bergonie, Bordeaux, France) and the PhD and postdoc session by Katrin Wiese (University of Amsterdam, the Netherlands) and Romain Amante (Friedrich Miescher Institute for Biomedical research, Basel, Switzerland).

## Abbreviations

3D: Three-dimensional
AI: Aromatase inhibitor
EMT: Epithelial–mesenchymal transition
ENBDC: European Network for Breast Development and Cancer
ER: Estrogen receptor
HMLE: Human breast epithelial
LTED: Long-term estrogen-deprived
TAD: topological associating domain
